# Durotaxis and Antidurotaxis Droplet Motion onto Gradient Gel-Substrates

**DOI:** 10.1021/acs.langmuir.4c02257

**Published:** 2024-08-06

**Authors:** Russell Kajouri, Panagiotis E. Theodorakis, Andrey Milchev

**Affiliations:** † Institute for Computational Physics, University of Stuttgart, 70569 Stuttgart, Germany; ‡ Institute of Physics, Polish Academy of Sciences, Al. Lotników 32/46, 02-668 Warsaw, Poland; ¶ Bulgarian Academy of Sciences, Institute of Physical Chemistry, 1113 Sofia, Bulgaria

## Abstract

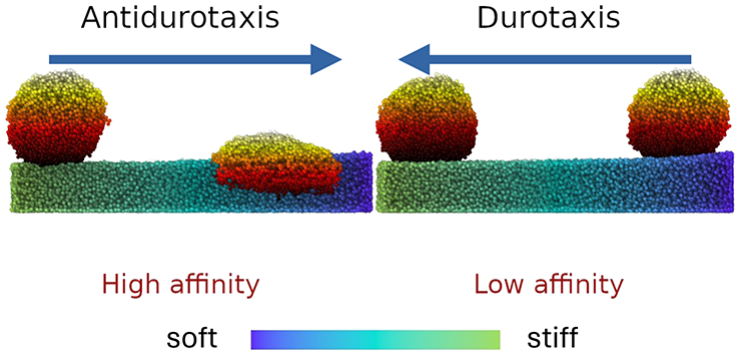

The self-sustained motion of fluids on gradient substrates is a spectacular phenomenon, which can be employed and controlled in applications by carefully engineering the substrate properties. Here, we report on a design of a gel substrate with stiffness gradient, which can cause the spontaneous motion of a droplet along (durotaxis) or to the opposite (antidurotaxis) direction of the gradient, depending on the droplet affinity to the substrate. By using extensive molecular dynamics simulations of a coarse-grained model, we find that the mechanisms of the durotaxis and antidurotaxis droplet motion are distinct, require the minimization of the interfacial energy between the droplet and the substrate, and share similarities with those mechanisms previously observed for brush substrates with stiffness gradient. Moreover, durotaxis motion takes place over a wider range of affinities and is generally more efficient (faster motion) than antidurotaxis. Thus, our study points to further possibilities and guidelines for realizing both antidurotaxis and durotaxis motion on the same gradient substrate for applications in microfluidics, energy conservation, and biology.

## Introduction

The autonomous motion of fluids on gradient substrates has been observed in various contexts, for example, in the case of microfluidics, microfabrication, coatings, energy conversion, and biology.^1−13^ Moreover, both the efficiency and the direction of motion can be controlled by carefully engineering the gradient of a substrate property. In the case of moving cells on tissues,^11,12,14−16^ their motion has been attributed to gradients in the stiffness of the underlying tissue, a phenomenon known as durotaxis. Inspired by biological systems, efforts to foster new possibilities of sustained motion on substrates with gradually changing properties along a certain direction have taken place, in view of the spectrum of possible applications in diverse areas. This also includes nano-objects of different type (e.g., fluids, nanosheets) on a wide range of different substrates, which have been studied in the context of theoretical and simulation work,^17−26^ as well as experiments.^27,28^

The exciting aspect of durotaxis is the autonomously sustained motion, that is no energy supply from an external source is required for setting in and sustaining the motion of the nano-object. While in connection with durotaxis, a gradient in the stiffness is responsible for the motion, such motion can actually be observed in other scenarios as well, for example, when the gradient reflects changes in the pattern of the substrate. Here, a characteristic example is rugotaxis, where a fluid motion is caused by a gradient in the wavelength characterizing a wavy substrate.^28,29^ Other examples include curvotaxis, that is motion attributed to curvature changes, such as that observed in the context of curved protein complexes at the cell.^30^ Further possibilities, include small condensate droplets that can move due to the presence of asymmetric pillars,^31^ three-dimensional (3D) capillary ratchets,^32^ or pinning and depinning effects at the three-phase contact line.^33^ Interestingly, in the case of capillary ratchets, the surface tension can play a role in determining the direction of motion, whether this is along or against the gradient.^32^ In addition, substrates with wettability gradients have been reported as a possibility for the autonomous motion of liquids,^34−36^ for example, due to corrosion,^13^ while long-range transport has been realized by using electrostatic^37,38^ or triboelectric charges.^39^ In the presence of an external energy source, motion is also possible, with characteristic examples being electrotaxis^40^ and thermotaxis.^41^ For example, in the latter case, the motion is caused by a temperature gradient that requires to be maintained along the substrate by means of an external energy source. Further examples of motion due to external sources include motion caused by electrical current,^42−45^ charge,^46−48^ or even simple stretching.^49^ Situations where droplets are chemically driven have also been reported in the literature,^50,51^ as well as droplets on vibrated substrates^52−55^ or wettability ratchets.^56−59^

Motivated by relevant experiments with liquid droplets,^27,28^ we have previously proposed and investigated by computer simulation various substrate designs that can cause a sustained droplet motion.^17,24,25,29^ More specifically, we have proposed two designs of brush substrates with stiffness gradient that can cause such motion either along or against the gradient direction.^24,25^ In the first design, the brush substrate had a constant density of grafted polymer chains.^24^ In this case, the stiffness gradient was a result of changes in the stiffness of the individual polymer chains along the gradient direction. We have found that the droplet can move toward areas of higher stiffness (durotaxis), where a larger number of contacts between the droplet and the substrate can be established, due to a lower substrate roughness in these areas. In the second design of a brush substrate, the grafted polymer chains were fully flexible and the stiffness gradient was imposed by changing the grafting density along a particular direction.^25^ In this case, the droplet could move toward softer parts of the substrate (antidurotaxis), establishing more pair contacts as it penetrated into the substrate. Interestingly, the latter antidurotaxis motion might share similarities with experiments of droplets on soft substrates with stiffness gradient, where droplet motion was also observed from stiffer toward softer areas of the substrate.^27^ Moreover, in this case, larger droplets seem to perform antidurotaxis motion more efficiently (faster), an effect that might not be attributed to gravity effects due to the weight of the droplet, as experiments were carried out for micrometer-sized water droplets, i.e., smaller than the capillary length (∼2.5 mm).

Thus, far, experimental substrates^11,12,14−16,27^ and simulation models^17,18,21,24,25^ have mostly demonstrated either durotaxis or antidurotaxis motion for a given substrate. Here, building upon our previous experience with durotaxis and antidurotaxis droplet motion onto brush substrates,^24,25^ we show that a novel gel substrate can demonstrate both antidurotaxis and durotaxis droplet motion depending on the type of liquid. To achieve this result, a gradient in the bonding stiffness between the gel chemical units is used in our model to create the stiffness gradient along a specific direction of the gel substrate. Furthermore, by means of extensive molecular dynamics (MD) simulations of a coarse-grained model, we elucidate the mechanisms for both the durotaxis and antidurotaxis motions and their efficiency for a range of parameters relevant for this substrate design. Interestingly, we observe similarities for these mechanisms with what we have previously seen for brush substrates.^24,25^ Thus, this may point to more universal features of such substrates that can cause durotaxis and antidurotaxis motion of fluids, and holds hope for the experimental realization of such substrates. In the following, we provide details of the system, simulation model and methodology. Then, we will present and discuss the obtained results, while we will draw the conclusions resulting from our investigations in the final section.

## Materials and Methods

**Figure 1:**
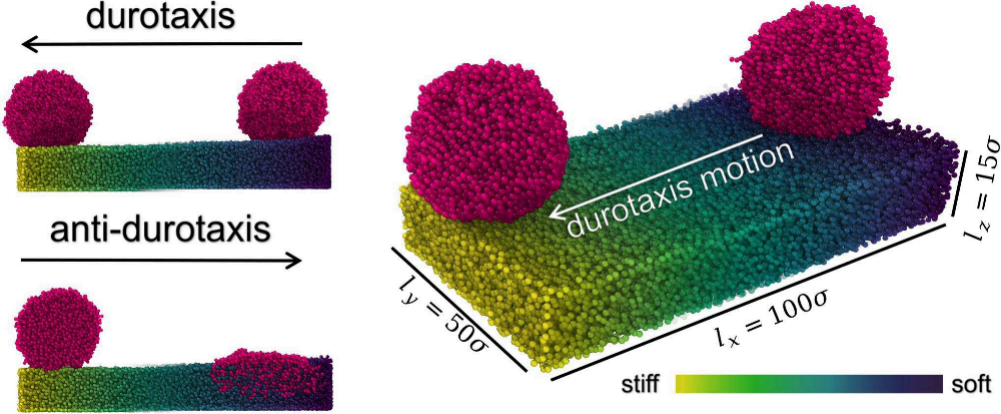
Typical conformations of the substrate after equilibration with the droplet on top at an initial and a final position with the direction of motion clearly indicated by the arrow for both the durotaxis (upper left and right panels) and the antidurotaxis cases (lower left panel). The stiffness gradient is visually represented by the color gradient with yellow reflecting areas of the highest stiffness and dark blue of the lowest. The dimensions of the gel substrate in the *x* (gradient direction), *y*, and *z* directions are *l*_*x*_ = 100 σ, *l*_*y*_ = 50 σ, and *l*_*z*_ ≈ 15 σ, respectively. The snapshot of the system was obtained using Ovito software.^60^

The gel substrate of this study is illustrated in Figure 1 with typical configurations of the droplet at the beginning and the end of successful durotaxis/antidurotaxis simulations. In particular, the droplet remains on the top of the substrate as it reaches the stiffest end of the substrate in the durotaxis case, while the droplet appears to penetrate into the substrate in the case of antidurotaxis motion as it reaches the softest end of the substrate. The length of the substrate in the direction of the gradient is *l*_*x*_ = 100 σ, where σ is the length unit. The gel substrate is supported by a smooth and unstructured substrate and consists of beads each initially placed at the positions of the vertices of a simple cubic lattice with unity lattice constant (expressed in units of σ) with harmonic interactions between beads reaching up to the second nearest neighbors. To realize the gradient in the substrate stiffness, the magnitude of these interactions (elastic constant, Γ_s_ in units of ε/σ^2^, where ε is the energy unit) linearly varies with the position *x* of the beads obtaining larger values toward the stiffer regions of the substrate (Figure 1), while the equilibrium length is set to 1.2 σ. The rate of change of Γ_s_ is 0.05 ε/σ^3^ at steps of 2 σ starting from an initial value of Γ_s_ = 0.5 ε/σ^2^ at the softest end of the substrate, thus implying Γ_s_ = 5 ε/σ^2^ at the stiffest end. Since this particular choice was proven to be optimal for carrying out our parametric investigation, our results will be based on this specific substrate setup. Once the substrate reached its equilibrium state by means of molecular dynamics simulation (further details will be provided below), a polymer droplet was first placed onto the softest and then the stiffest part of the substrate to examine the direction of motion (antidurotaxis or durotaxis). Once, the direction of motion was identified, the decision was taken onto which end of the substrate the droplet should be placed and an ensemble of simulations were carried out for each set of parameters.

Nonbonded interactions between particles (beads) in the system are based on the Lennard-Jones (LJ) potential, expressed by the relation
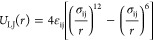
1Here, *r* is the distance between any pair of beads, with indices i and j in eq 1 reflecting the type of bead, namely “d” for the droplet and “g” for the gel substrate. The size of the beads is the same, namely σ_ij_ = σ. Attractive interactions between the gel and the droplet beads as well as among the droplet beads are used by choosing a cutoff of *r*_c_ = 2.5 σ for the LJ potential, while an athermal model is used for the interactions among the gel beads. The strength of LJ interactions between the droplet beads is set to ε_dd_ = 1.5 ε. Different choices are considered for the interaction strength between the polymer droplet and the gel substrate, namely ε_dg_ = 0.3–1.0 ε, thus in practice controlling the affinity of the droplet to the substrate. Finally, the droplet consists of fully flexible polymer chains to avoid evaporation effects, which may also further complicate our analysis. Hence, the vapor pressure is sufficiently low.^61^ In particular, the droplet consists of polymer chains with length *N*_l_ = 10 beads each, while the total size of the droplet is 8000 beads. To bind the beads together in each polymer chain of the droplet a harmonic potential was used with elastic constant 1000 ε/σ^2^ and equilibrium length σ.

To control the temperature of the system, *T* = ε/*k*_*B*_ (*k*_*B*_ is Boltzmann’s constant), the Nosé–Hoover thermostat was applied,^62,63^ as implemented in the HOOMD-Blue package (version 2.9.7).^64^ The integration time step was set to 0.005 τ, where  is the natural MD time unit. For every set of parameters, we perform five simulation experiments with different initial conditions (i.e., changing the random seed for generating the initial velocities of the system) to statistically collect data for the analysis of properties. Finally, each simulation run lasts a total of 50 × 10^6^ time steps, which was deemed long enough for drawing reliable conclusions on the possibility of observing the droplet motion and carrying out the necessary analysis of the relevant properties.

**Figure 2:**
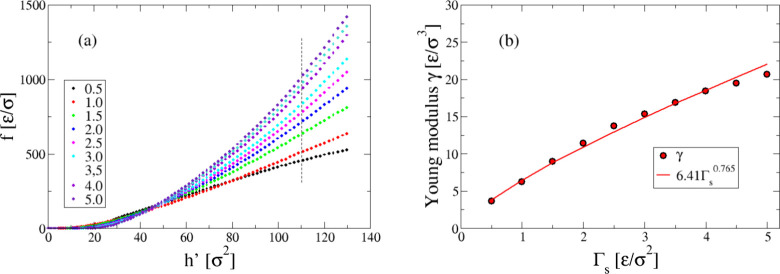
(a) Force *versus h*′ = *αh*^3/2^ for different choice of the elastic constant for the harmonic interaction between gel beads, Γ_s_, as indicated. According to eq 2, the slope yields the Young modulus, γ, for each case. Here, data refer to substrates with no stiffness gradient. Hence, Γ_s_ is constant along the substrate for each case. The maximum value used for the fit is indicated by the vertical dashed line. (b) Young modulus, γ, for each substrate without gradient versus Γ_s_. The solid, red line corresponds to a power-law fit.

Before presenting our durotaxis and antidurotaxis experiments and their analysis, we take a step back to analyze the stiffness of the substrate and see how this varies with the strength of the interactions between beads used to create the gradient. To perform our analysis, we consider a nanoindenter that slowly impinges onto the gel substrate without gradient, as has been done in a previous study in the case of protein fibrils.^65^ By recording the total force of the substrate beads on the nanoindenter, the Young modulus, γ, of the gel substrate can be determined similarly to an empirical technique used to estimate the Young modulus in atomic-force-microscopy (AFM) nanoindentaion experiments. The Young modulus of the nanoindenter is infinite and we therefore define each system in the limit of the Hertzian theory.^66^ The indenter is a sphere with a curvature radius *R*_ind_ that slowly impinges onto the gel substrate with a velocity *u*_ind_. Here, this velocity was the same in all nanoindentation exeriments, i.e., data were collected every 5 × 10^3^ MD time steps for a total trajectory length of 5 × 10^5^ time steps with a time step of 0.005 τ. Then, the nanoindentation force, *f*, is defined by the Hertz relation
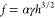
2where *h* is the penetration or nanoindentation length, γ Young modulus, and
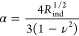
3In our simulation experiments, the radius of the nanoindenter was *R*_ind_ = 5 σ and the maximum penetration depth *h*_max_ = 10 σ. ν is the Poisson coefficient, in our case taken as 0.5, which corresponds to a homogeneous deformation on the *x* – *y* plane. Then, the Young modulus, γ, can be determined by calculating the slope of the curves of Figure 2a for each gel substrate without gradient but with a different value of the harmonic elastic constant, Γ_s_. By plotting the obtained Young’s moduli as a function of Γ_s_ (Figure 2b), we conclude that increasing Γ_s_ indeed results in stiffer gel substrates. By attempting to fit a power-law function on these data, we obtained an exponent of about 3/4 for the relation between γ and Γ_s_.

## Results and Discussion

**Figure 3:**
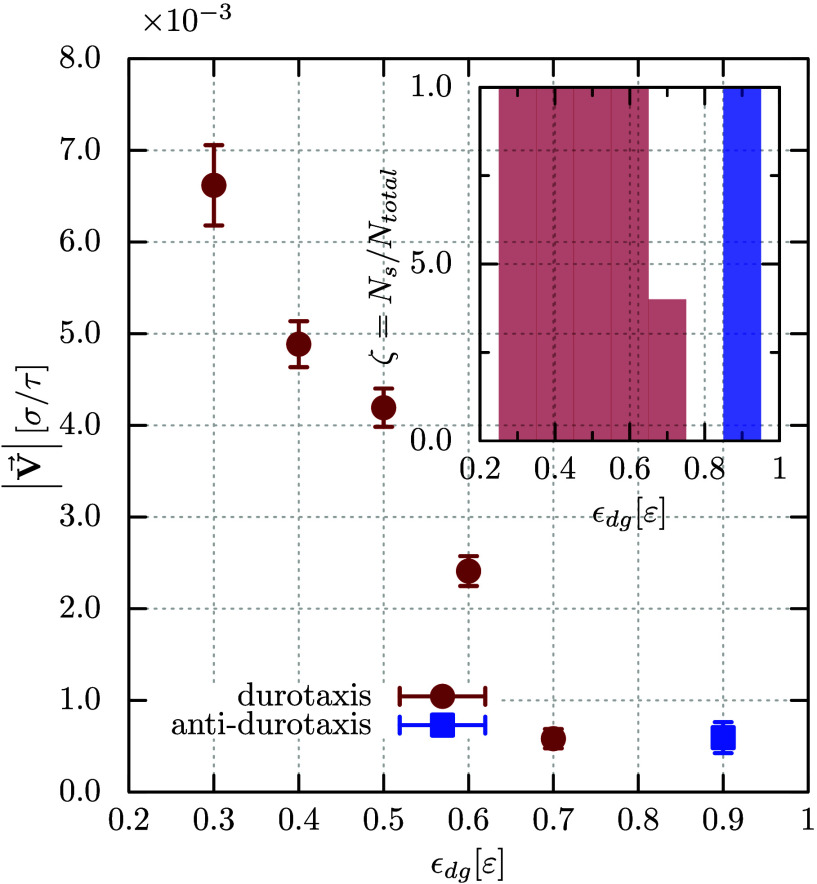
Average speed of the droplet as calculated from successful durotaxis/antidurotaxis experiments (*N*_s_ is the number of the successful cases) from a total ensemble of five (*N*_total_ = 5) trajectories for each case, as indicated. Inset shows the probability, ζ = *N*_s_/*N*_total_, of the droplet moving from one side of the substrate to the other. This probability for antidurotaxis cases is illustrated by purple bars, while that for the durotaxis cases by brown color. For ε_dg_ < 0.8 ε durotaxis is observed, while antidurotaxis was recorded for ε_dg_ = 0.9 ε. For ε_dg_ = 0.8 ε, only partial droplet motion was observed from each trajectory and therefore no successful cases are reported in the plot.

Given the constant gradient maintained in each of our simulation experiments, which is optimally chosen to facilitate our properties exploration, the first aspect of our research concerns the possibility of causing durotaxis or antidurotaxis motion and the probability of such motion for a range of droplet–substrate affinities. To address this issue, a droplet is placed either on the softest or the stiffest part of the substrate and the outcome of the simulation is monitored. Figure 3 visually summarizes our conclusions. For values ε_dg_ < 0.2 ε, the interaction between the droplet and the gel substrate is weak. Hence, in this case the droplet detaches from the substrate due to the thermal fluctuations and this case deserves no further consideration here. Durotaxis motion takes place when 0.2 ε < ε_dg_ < 0.8 ε. For this range of affinity strength between the droplet and the substrate, we observe that the droplet moves from softer to stiffer parts of the gel substrate covering its full length in the *x* direction, a manifestation of successful durotaxis motion for the droplet. While for 0.3 ε ≤ ε_dg_ ≤ 0.6 ε the probability that the droplet successfully moves from the softest to the stiffest side of the substrate is 1.0 as calculated from an ensemble of five different trajectories for each affinity case, this probability becomes less than unity when ε_dg_ = 0.7 ε. Moreover, we were able to only detect partial motion along the substrate, when ε_dg_ = 0.8 ε, reporting threrefore this case as unsuccessful. This provides a first indication that the droplet motion may become less effective for larger values of ε_dg_. Indeed, this is corroborated by monitoring the average velocity of the droplet for different values ε_dg_ (Figure 3), which clearly indicates that an increased affinity between the droplet and the substrate will lead to a smaller average durotaxis velocity. Further increase of the affinity, namely ε_dg_ = 0.9 ε, leads to successful antidurotaxis motion. In this case, the droplet reached the softest part of the gel substrate and the recorded average velocity was of the same magnitude as in the durotaxis case with ε_dg_ = 0.7 ε. Finally, antidurotaxis motion for ε_dg_ = ε was observed, but in this case the droplet was not able to cover the full distance from the one to the other side of the gel substrate for any of our five trajectories and therefore this case was considered unsuccessful, as was the case of partial durotaxis droplet motion for ε_dg_ = 0.8 ε. The above observations may allow us to conclude that both durotaxis and antidurotaxis motions are possible on the same substrate. Since this takes place by varying the droplet–substrate affinity in our simulation, we may argue that the direction of motion eventually depends on the choice of liquid for the droplet. Also, durotaxis motion on gel substrates is overall more efficient than the antidurotaxis motion, especially when the droplet–substrate affinity is lower.

**Figure 4:**
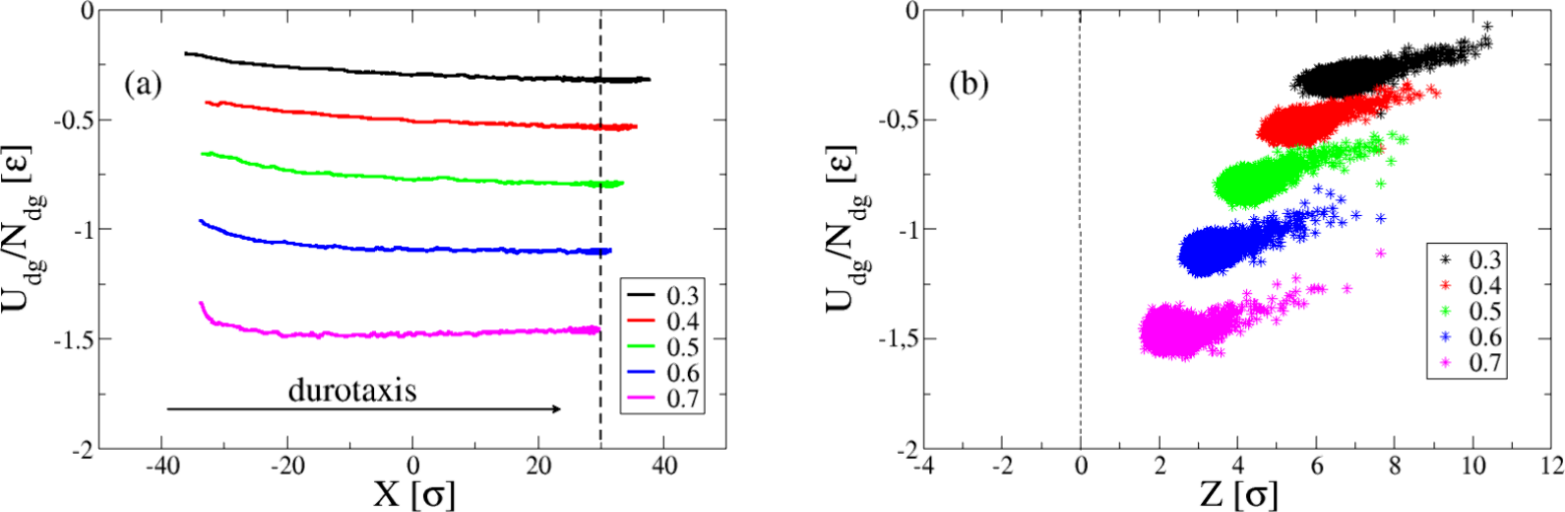
(a) Interfacial energy of the droplet normalized by the number of substrate–droplet bead pairs as a function of the *X* coordinate of the center-of-mass of the droplet in the *x* direction for a range of different durotaxis cases with different ε_dg_, as indicated. The vertical dashed line indicates the *X* position for the center-of-mass considered for determining the successful translocation of the droplet toward the stiffest end of the substrate. (b) The normalized interfacial energy is plotted against the coordinate of the center-of-mass of the droplet in the *z* direction. The vertical dashed line denotes the position of gel’s surface, calculated by the inflection point of the density profile of the gel, as is done in our previous study.^25^

As in our previous studies,^17,24,25,29^ we attempted to identify the driving force for both antidurotaxis and durotaxis cases. *X* in Figure 4 indicates the coordinate of the center-of-mass of the droplet in the *x* direction with the zero value corresponding to the center of the gel substrate. *Z* is the coordinate of the center-of-mass of the droplet in the *z* direction with the zero indicating the position of the substrate boundary, which was determined through the inflection point in the density profile of each substrate as done in our previous work.^25^ Moreover, the peculiarities of the gel–droplet interface have been explored recently in detail.^67^ On the basis of our analysis for the durotaxis cases, we observe that the interfacial energy between the droplet and the substrate decreases as a function of the center-of-mass position of the droplet in both the *x* (Figure 4a) and the *z* directions (Figure 4b), which suggests that the droplet establish a larger number of contacts with the gel as it moves along the substrate (see also Movie S1 in the Supporting Information). As a result, the droplet is more strongly attracted by the gel as it moves toward the stiffer parts, which results in a decrease in the position *Z* of the center-of-mass of the droplet, but with the droplet however remaining on top of the substrate. Moreover, we observe that the slope in the energy reduction of the interfacial energy as a function of the position *X* of the center-of-mass of the droplet is larger for smaller values of the attraction strength ε_dg_ (Figure 4a), which reflects the conclusions relating to the average velocity of the droplet presented in Figure 3, that is a lower adhesion of the droplet to the gel substrate offers a more efficient (in terms of droplet speed) durotaxis motion. This motion mechanism of the droplet shares similarities with the durotaxis motion previously observed on brush substrates,^24^ where the droplet moves to the areas of smaller surface fluctuations of the substrate, that is substrate parts of lower roughness.

**Figure 5:**
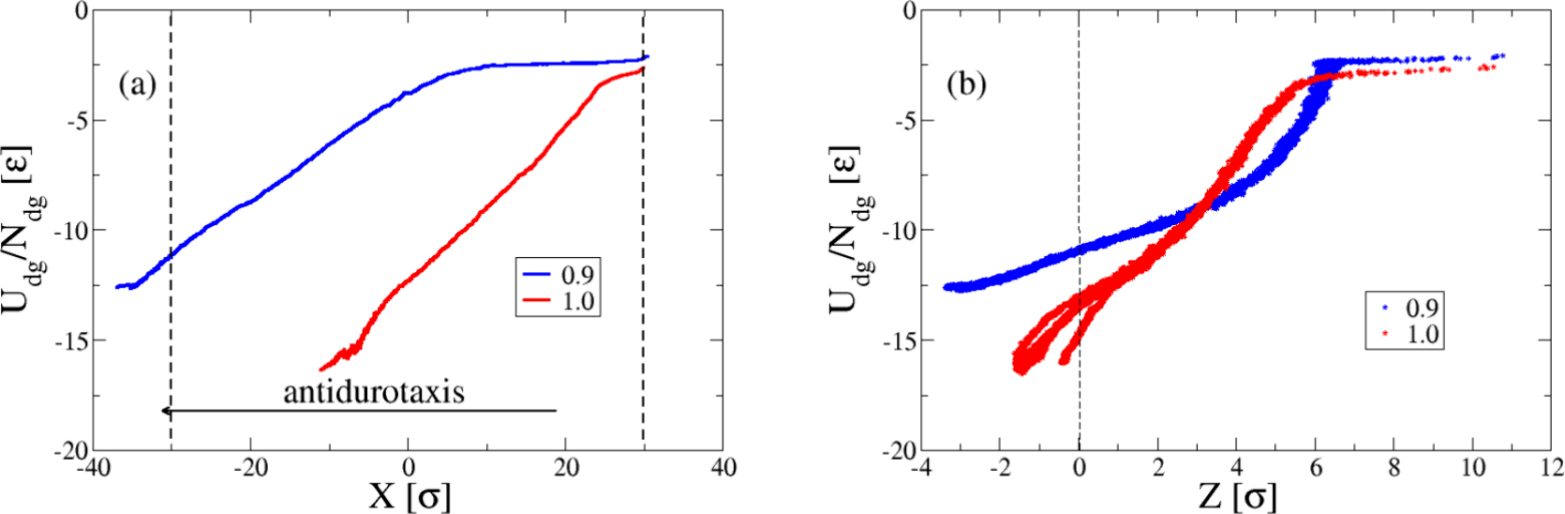
(a) Interfacial energy of the droplet normalized by the number of substrate–droplet bead pairs as a function of the *X* coordinate of the center-of-mass of the droplet in the *x* direction for a range of different antidurotaxis cases with different ε_dg_, as indicated. (b) The interfacial energy is plotted against the coordinate of the center-of-mass of the droplet in the *z* direction.

The results of Figure 4 for the durotaxis cases can be compared with those for the antidurotaxis cases presented in Figure 5. Notably, we observe that the interfacial energy is much more reduced for the antidurotaxis cases in comparison with the durotaxis ones. More importantly, we also see that the droplet penetrates deeper into the substrate in the case of antidurotaxis droplet motion and the center-of-mass of the droplet eventually lies below the top of the substrate as the antidurotaxis motion completes (see also Movie S2 of the Supporting Information). This mechanism is therefore more similar to the one observed in the case of antidurotaxis motion for brush substrates with gradient in the grafting density of the polymer chains.^25^ In this case, the minimization of the interfacial energy was due to the penetration of the droplet into the brush substrate. For this reason, the droplet motion is much less efficient than that in the case of durotaxis simulations, since the droplet faces a larger resistance in carrying out the motion along the substrate by bypassing the gel beads.

**Figure 6:**
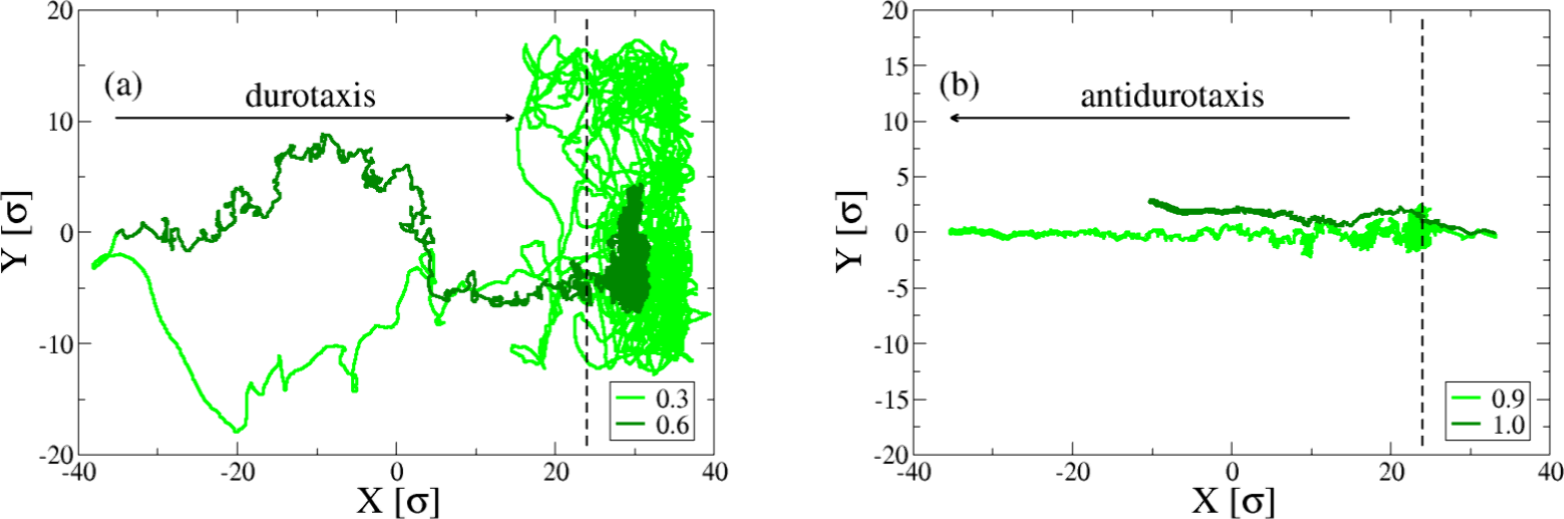
Typical trajectories for (a) durotaxis and (b) antidurotaxis cases for values of ε_dg_, as indicated. The points of the trajectory are collected by tracking the center-of-mass of the droplet on the *x* – *y* plane.

Finally, we monitored the trajectories of the center-of-mass of the droplet onto the *x* – *y* plane (Figure 6). A different behavior of the droplet motion is observed between durotaxis and antidurotaxis cases. In particular, we see that the droplet motion is more influenced by thermal fluctuations as indicated by the lateral motion in the *y* direction in the case of durotaxis (Figure 6a). The droplet clearly initially moves at a higher instantaneous speed toward the stiffer areas and then slightly slows down. This pattern of motion is observed for both the lowest and the highest affinity between the droplet and the substrate, which may indicate that the affinity might play a lesser role in determining the exact trajectory of the particle. The weakening of the gradient effect on the droplet velocity as the droplet reaches the ever stiffer parts of the substrate has been thus far observed in all previous durotaxis/antidurotaxis studies.^17,24,25^ In the case of antidurotaxis experiments (Figure 6b), the droplet appears to only move in the *x* direction with minimal lateral (diffusive) motion in the *y* direction, which may suggest that the motion in this case is dominated by the droplet–substrate interactions. This takes place to a larger degree as the droplet moves to the softer parts of the substrate. Hence, we can see that the droplet motion fundamentally differs in the case of antidurotaxis and durotaxis cases, with the antidurotaxis motion providing a more certain path for the trajectory of the droplet moving along the substrate during the simulation experiments.

## Conclusions

In this study, we have proposed and investigated a novel substrate design based on a gel material. Importantly, we have been able to demonstrate that durotaxis and antidurotaxis motion of a droplet is possible on the same substrate and the direction of motion only depends on the fluid. To our knowledge, this is the first time that this possibility is realized for gel substrates. As in the case of durotaxis onto brush substrates,^24,25^ we have found that the minimization of the interfacial energy between the droplet and the substrate is the dominant driving force responsible for the motion of the droplet. This takes place by the substantial penetration of the substrate by the droplet in the case of antidurotaxis or the droplet motion toward areas with smaller surface fluctuations on the top of the gel in the case of durotaxis. As a result, the trajectories of the droplet motion appear to be more diffusive in the durotaxis cases than in the antidurotaxis cases, where in the latter the droplet motion is hindered by the gel units. Moreover, recent experiments^68^ have reported on the spontaneous droplet motion on soft, gel substrates with stiffness gradient created by varying the degree of cross-linking in the gel. In this case, results have pointed out to the minimization of the interfacial energy between the substrate and the droplet as the driving force for the durotaxis motion of the droplet, as in the case of simulation experiments here and in previous studies.^17,24,25^ We have also found that durotaxis takes place for a wide range of droplet–substrate affinities with lower affinities leading to more efficient durotaxis motion, while fully successful antidurotaxis motion has only been observed for a high value of droplet–substrate affinity.

Our study provides further evidence that both durotaxis and antidurotaxis motion can be realized on the same gel substrate. Thus, we anticipate that our work highlights the new venues of possibilities in the autonomous motion of fluids based on gradient gel-substrates and provides insights into the motion of droplets driven by stiffness gradients, thus enhancing our understanding of similar phenomena, encountered in nature.

## Supporting Information Available

Movie 1 illustrates the droplet durotaxis motion (ε_dg_ = 0.3 ε) (MP4)
Movie 2 illustrates the droplet antidurotaxis motion (ε_dg_ = 0.9 ε) (MP4)
